# Select and Sequence of a Segregating Sugar Beet Population Provides Genomic Perspective of Host Resistance to Seedling *Rhizoctonia solani* Infection

**DOI:** 10.3389/fpls.2021.785267

**Published:** 2022-01-13

**Authors:** Paul Galewski, Andrew Funk, J. Mitchell McGrath

**Affiliations:** ^1^United States Department of Agriculture – Agricultural Research Service (USDA-ARS) Northwest Irrigation and Soils Research Laboratory, Kimberly, ID, United States; ^2^Department of Plant, Soil, and Microbial Science, Plant Breeding, Genetics, and Biotechnology Program, Michigan State University, East Lansing, MI, United States; ^3^United States Department of Agriculture – National Institute of Food and Agriculture (USDA-NIFA) Institute of Food Production and Sustainability, Kansas City, MO, United States; ^4^United States Department of Agriculture – Agricultural Research Service (USDA-ARS) Sugar Beet and Bean Research Unit USDA-ARS, East Lansing, MI, United States

**Keywords:** *Beta vulgaris*, sugar beet, Rhizoctonia resistance, synthetic populations, gene discovery

## Abstract

Understanding the genetic basis of polygenic traits is a major challenge in agricultural species, especially in non-model systems. Select and sequence (SnS) experiments carried out within existing breeding programs provide a means to simultaneously identify the genomic background of a trait while improving the mean phenotype for a population. Using pooled whole genome sequencing (WGS) of selected and unselected bulks derived from a synthetic outcrossing sugar beet population EL57 (PI 663212), which segregates for seedling rhizoctonia resistance, we identified a putative genomic background involved in conditioning a resistance phenotype. Population genomic parameters were estimated to measure fixation (*He*), genome divergence (*F*_*ST*_), and allele frequency changes between bulks (DeltaAF). We report on the genome wide patterns of variation resulting from selection and highlight specific genomic features associated with resistance. Expected heterozygosity (*He*) showed an increased level of fixation in the resistant bulk, indicating a greater selection pressure was applied. In total, 1,311 biallelic loci were detected as significant F_ST_ outliers (*p* < 0.01) in comparisons between the resistant and susceptible bulks. These loci were detected in 206 regions along the chromosomes and contained 275 genes. We estimated changes in allele frequency between bulks resulting from selection for resistance by leveraging the allele frequencies of an unselected bulk. DeltaAF was a more stringent test of selection and recovered 186 significant loci, representing 32 genes, all of which were also detected using F_ST_. Estimates of population genetic parameters and statistical significance were visualized with respect to the EL10.2 physical map and produced a candidate gene list that was enriched for function in cell wall metabolism and plant disease resistance, including pathogen perception, signal transduction, and pathogen response. Specific variation associated with these genes was also reported and represents genetic markers for validation and prediction of resistance to Rhizoctonia. Select and sequence experiments offer a means to characterize the genetic base of sugar beet, inform selection within breeding programs, and prioritize candidate variation for functional studies.

## Introduction

The characterization of resistance sources for the genetic improvement of beet (*Beta vulgaris*) is a long-standing challenge. The USDA sugar beet germplasm is enriched for important traits such as resistance to disease and adaptation to local production regions. The pedigrees of this material suggest many of these traits can be traced back to wide hybridizations between sugar beet and wild beet (*Beta maritima*) (Doney, 1995; Panella et al., 2015). Beet is an outcrossing species, wind pollinated and generally self-incompatible. As a result, beet populations are highly heterozygous. Historically, crop improvement has been carried out via recurrent phenotypic selection and sib mating (Doney and Theurer, 1978). Commercial sugar beets are hybrid, and the production of hybrid seed relies on a narrow but well characterized genetic base where the frequencies of cytoplasmic male sterility (CMS) and CMS restorers are well understood. The need to maintain the genetic backgrounds required for hybrid seed production while breeding for multiple disease resistance traits, local adaptation, and yield makes the utilization of novel genetic variation a slow and resource-intensive process. Beet reference genomes and sequencing technologies have increased our ability to characterize genome variation within diverse beet lineages and breeding lines (Funk et al., 2018; Galewski and McGrath, 2020). The use of whole genome sequencing (WGS) to inform traditional beet breeding programs provides a system for “select and sequence” (SnS) experiments. These methods are a powerful tool for detecting the genetic basis of phenotypic selection in experimental populations (Schlötterer et al., 2015; Burghardt et al., 2018) and is an efficient way to prioritize candidate variation for functional studies and marker validation in the future (Burny et al., 2020).

Advances in genomic resources, population genomic methods and experimental design have improved the process of gene discovery in agricultural species. Reference genomes provide an anchor to link genetic variation with coding sequences underlying phenotypic variation (Hufford et al., 2012). Accurate gene models determined from ab-initio gene prediction, transcript evidence, and gene ontology can help to infer gene function and provide hypotheses for biological mechanisms (Salzberg, 2019). The genome EL10.2 is a contiguous chromosome level assembly (McGrath et al., 2020) with high homology to other beet reference genomes such as RefBeet (Dohm et al., 2014). Reference genomes have increased our ability to rapidly catalog and compare variation within and between populations using genome scans (Nielsen et al., 2005), bulk segreant analysis (BSA) (Michelmore et al., 1991) and mapping by sequencing approaches (Schneeberger et al., 2009). WGS has also been used to provide a complete picture of genetic variation within a population including structural variants (SV) and presence-absence variation (PAV) (Pinosio et al., 2016; Wang et al., 2018). Recent research has demonstrated the impact of SV and PAV on important phenotypic variation observed between cultivars and adaptative trait variation including disease resistance (Zhou et al., 2019; Hämälä et al., 2021). SnS experiments show potential to detect genetic variation linked to selection and adaptation in experimentally generated populations (Schlötterer et al., 2015). Additionally, numerous methods to detect positive selection within populations have been established (reviewed in Weigand and Leese, 2018) which facilitates the adoption of SnS experiments within breeding programs for agricultural crops such as beet.

Phenotypic variation in beets is often measured at the level of the population due to difficulties inbreeding and fixing variation in single plants, fostering comparisons between populations rather than comparisons between individuals. Pooled sequencing of populations is an effective way to capture and compare causal genetic variation in beet, either by partitioning variation according to phenotypes within a segregating population, or by comparing different populations. The utility of pooled data has been demonstrated (Schlötterer et al., 2014) and can provide accurate estimates of allele frequency (Lynch et al., 2014) and population genetic parameters (Ferretti et al., 2013). Pooled sequencing increases the effective number of assayed recombination events, improving our ability to resolve causal variation vs. traditional mapping approaches. In beets, pooled sequencing has been utilized to understand how diversity is distributed in crop type lineages (Galewski and McGrath, 2020) and within selected breeding populations (Ries et al., 2016). The later research used pooled data and a mapping by sequencing approach to discover causal variation associated with hypocotyl color, a monogenic trait. Our hypothesis is that pooled sequencing could also be effective for resolving polygenic traits with continuous phenotypes, which are more difficult to detect with traditional marker-based approaches. For example, disease resistance traits have been a major target of sugar beet breeding for more than a century and the application of pooled sequence data to inform polygenic traits such as Rhizoctonia resistance is warranted.

*Rhizoctonia solani* Kühn is a soil borne pathogen which can cause seedling dampening off and crown and root rot, both of which can severely impact sugar yield for growers (Gaskill et al., 1970). Various management practices are used to mitigate *R. solani* infection in the field and maintain crop profitability. This includes crop rotation, seed treatments and fungicide applications (Bolton et al., 2010). Genetic resistances have been identified but appear to be derived from relatively few sources (Panella, 2005) which highlights the need for identifying new sources. Unfortunately, genetic resistance to rhizoctonia is poorly characterized, and while no single germplasm source can be attributed to Rhizoctonia resistance in beet, the long history of selection for resistance to crown and root rot from the USDA-ARS Ft. Collins, CO, United States germplasm enhancement program likely represents the major resistance source in commercial materials. Seedling resistance was identified from these materials and from the USDA-ARS East Lansing, MI, United States germplasm enhancement program (Panella et al., 2015). Early reports describe resistance as polygenic with many small-effect alleles (Hecker and Ruppel, 1975). Some major resistance quantitative trait loci (QTL) have been described in greenhouse studies (Lein et al., 2008) but the added complexity of field conditions and year effects (Strausbaugh et al., 2013a), host growth stage (Nagendran et al., 2009; Liu et al., 2019), cultivar and pathogen interactions (Strausbaugh et al., 2013b), and confounding infections from bacterial pathogens (Strausbaugh et al., 2013a) suggest many genes contribute to rhizoctonia resistance in sugar beet. Other research suggests the involvement of additional compounds and proteins in rhizoctonia resistance, such as reactive oxygen species (Taheri and Tarighi, 2010), polygalacturonase-inhibiting proteins (Li and Smigocki, 2018), and major latex protein-like proteins (Holmquist et al., 202). Newly sequenced rhizoctonia genomes have further detailed the complexity of host-pathogen interactions resulting from different anastomosis groups, putative genes, enzymes, and effectors molecules (Wibberg et al., 2016).

This research is focused on understanding plant host resistance to seedling Rhizoctonia by sequencing bulks of phenotypically distinct individuals derived from a synthetic outcrossing population, EL57 (PI 663212). Using WGS, existing reference genomes, and selection for resistance we highlight a genomic background associated with seedling Rhizoctonia resistance. In identifying the genetic determinants underlying resistance we show how these methods can be used to characterize polygenic traits in beet (*B. vulgaris)*, inform future experiments, and provide genetic solutions to long standing challenges faced by sugar beet producers.

## Materials and Methods

### Populations and Sequencing

The population EL57 is a unique synthetic population combining mostly Eastern US germplasm traits in a self-fertile genetic background, and is diploid, multigerm, and biennial. EL57 is a very broad genetic base diploid combining genetics of 660 mother roots with the unique feature that 98% of the 133 parental used lines are self-fertile due to a dominant gene (Sf) introgressed from C869 (PI 628754) or C869 CMS (PI 628755). 21% of the parents were derived from C869 CMS and thus have the S-cytoplasm. Male sterility, both nuclear male sterility from C869 and CMS, was used to capture pollen from open-pollinated increases of a wide variety of pollinators from 1997 through 2007. Traits expected to be segregating in the population include Aphanomyces seedling disease and Cercospora leaf spot resistances contributed by sugar beet germplasms SP7622 (aka SP6822, 20% of original pollinators), USH20 (8% of original pollinators), and SP85303 (PI 590770, 6% of original pollinators), Rhizoctonia resistance derived from EL51 (PI 598074, 13% of original pollinators), curly top and rhizomania resistance selections from C931 (PI 636340) and EL0204 (PI 655951) (5% of original pollinators), a series of Aphanomyces resistant or salt-tolerant germination breeding lines and selections (derived from PI 165485, PI 271439, PI 518160, PI 546409, PI 562591, PI 562599, and PI 562601) (20% of original pollinators in total), a series of 17 nematode resistant breeding lines from the Salinas, CA USDA-ARS breeding program (13% of original pollinators), and a mixture of released and unreleased breeding lines derived from high sucrose, smooth-root selections (23% of original pollinators).

EL57 was planted in the SVREC seedling Rhizoctonia nursery on May 15, 2017, as a large selection block of 161 plots and inoculated with Rhizoctonia isolate RG2-2 on June 6. Seven plots of EL57 were not inoculated as a control. Approximately 8 weeks later leaves were harvested from non-inoculated and inoculated plots, representing three bulks. Resistant and susceptible bulks were chosen from the inoculated plots, and an unselected bulk was taken from non-inoculated plots. Leaf material from 25 plants was harvested and pooled for each of the three bulks. Pooled leaf material was homogenized, and DNA was extracted using the Macherey-Nagel NucleoSpin Plant II Genomic DNA extraction kit (Bethlehem, PA). One microgram of DNA for each population was submitted to Admera Health, LLC, where NGS libraries were constructed using TruSeq bar-code adapters. The sequencing reactions were carried out on the Illumina Hi-Seq 2500 in a 2 × 150 bp paired-end format with a target coverage of 80x relative to the predicted 758 Mb genome size of beet (Arumuganathan and Earle, 1991). Post-sequencing read quality was assessed using FastQC (Andrews, 2010). Library bar-code adapters were removed and reads were trimmed according to a quality threshold using TRIMMOMATIC (Bolger et al., 2014) invoking the following options (ILLUMINACLIP:adapters.fa:2:30:10 LEADING:3 TRAILING:3 SLIDINGWINDOW:4:15 MINLEN:36). These filtered reads were used for downstream analysis.

#### Alignment and Variant Detection

Reads from each population were aligned to the *B. vulgaris* reference genome assembly EL10.2 (McGrath et al., 2020) using BWA mem (Li, 2013). The resulting alignment files were sorted and merged using SAMtools (Li et al., 2009). Variants for each population were called simultaneously on all three populations using the program Freebayes (Garrison and Marth, 2012). Variants were filtered for mapping quality, number of variants detected and depth across sites. After the initial variant detection step, the vcf file was filtered for genotype quality, GQ ≥ 20, and read depth, *N* < 300. Variants were then partitioned into those that were detected as biallelic and those that were multi-allelic. The biallelic sites were used for the estimation of population genetic parameters and the multiallelic sites were retained for consequence on phenotype after significant regions were determined. In addition, structural variants were cataloged in each sample using the program Manta (Chen et al., 2016).

#### Genome Divergence—Allele Frequency, F_ST_ and DeltaAF

A python program was used to count alleles for all biallelic variants within the three populations. Allele frequency was estimated for the resistant, susceptible, and unselected EL57 populations. Population genetic parameters were estimated using the allele frequency within each population such that (*p* + *q* = 1). The variable *p* was designated as the reference allele of the EL10.2 reference genome and *q* as the alternate state. The degree of fixation was estimated at all biallelic sites using the expected heterozygosity (*He)* or *2pq.* Global levels of fixation with respect to selection were calculated as the average of *He* across all sites. F_ST_ was used to calculate differentiation between the resistant and susceptible populations at each locus (Eq. 1). The parameter F_ST_24 was estimated by calculating F_ST_ within a sliding window of 25 biallelic variant sites, or 12 variant sites flanking a single biallelic locus across the genome. Significant regions along chromosomes were determined by loci that showed a significant F_ST_ value at a given locus. Significance thresholds were defined by *P*-values < 0.01, calculated using the empirical distribution of all F_ST_ values.


(1) $$F_{ST}=\frac{\sigma_{s}^{2}}{\sigma_{T}^{2}}=\frac{\sigma_{s}^{2}}{\bar{p}\left(1-\bar{p}\right)}$$

*Equation 1: shows F_ST_ is defined as the ratio of variance in allele frequency of the subpopulation (s) relative to the total population (t), where p is the allele frequency of allele (p).*

Delta allele frequency was calculated by using a series of Boolean operators to determine the loci which pass allele frequency thresholds in selected populations relative to the unselected population. This provided a null distribution from which to derive the genomic locations of large changes in allele frequency with respect to selection for resistance.


(2) $$\begin{array}{c} DeltaAF\left(RS\right)=max\left(AFR,AFS\right)—min\left(AFR,AFS\right)\\ DeltaAF\left(RW\right)=max\left(AFR,AFS\right)—min\left(AFR,AFS\right)\\ DeltaAF\left(SW\right)=max\left(AFR,AFS\right)—min\left(AFR,AFS\right)\\ DeltaAF=\left(DeltaAF\left(RS\right)\text{ > }0.8;DeltaAF\left(SW\right)\text{ < }0.15\right) \end{array}$$

*Equation 2: determines sites where the relative change in allele frequency between resistant and susceptible bulks is > 0.8 and the allele frequency change between susceptible and unselected bulks is low, >0.15.*

#### Genome Visualization

Visualization of significant genomic regions was carried out by plotting F_ST_24 along chromosomes with a density plot of all significant F_ST_ values. Python was used for the manipulation of data sets and estimation of population genetic parameters, while R libraries were used for plotting the final data matrixes. DeltaAF was also plotted across the genome, highlighting only those regions where divergence in allele frequency between the resistant and susceptible bulk was high and the susceptible and unselected population was low. Regions along the chromosome of high significance were determined by investigating a significant locus and searching within a 50 kb window upstream to determine the size and significance of a region with respect to selection. All regions with significant F_ST_ or DeltaAF were visualized using R. The density and distribution of variation was also considered by plotting data relative to the physical map provided by the EL10.2 genome assembly along with EL10 gene models and annotations. SNPeff (Cingolani et al., 2012) was used to annotate variants based on physical position and determine functional consequences in terms of protein coding changes.

#### Determination of Resistance Genes Involved in Resistance to *Rhizoctonia solani*

A combination of statistical analysis and genome resources were leveraged to identify targets within or adjacent to significant regions across the genome (e.g., F_ST_, F_ST_24, and DeltaAF). Determination of putative gene loci involved in resistance were based on all previous analysis as well as significant homology to functional validated candidates in other species. Markers were derived for use in predicting seedling rhizoctonia resistance by extracting significant variation from .vcf files.

## Results

**FIGURE 1:**
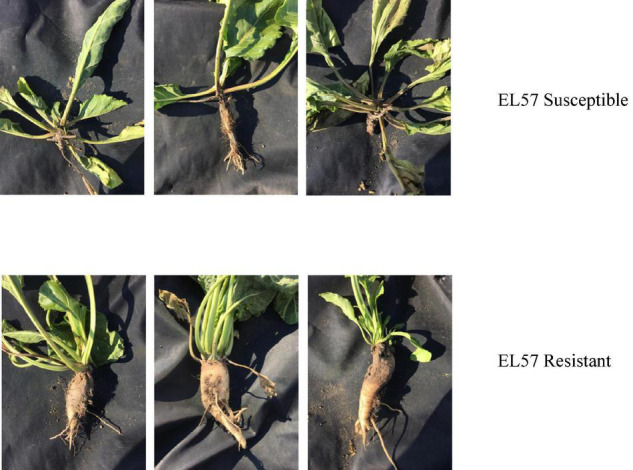
Phenotypic selection of EL57 bulks segregating for resistance to seedling Rhizoctonia.

Nearly 3,500 plants from the synthetic, outcrossing sugar beet population EL57 (PI 663212) were sown, inoculated with *Rhizoctonia solani* isolate RG2-2, and allowed to grow for 8 weeks before being evaluated for seedling dampening off. Rhizoctonia symptoms became progressively worse throughout the growing season: average stand counts prior to inoculation were 21.3 plants/plot (*SD* = 3.33) and post-inoculation at the end of the season (October 17) were 4.9 plants/plot (*SD* = 1.74), or approximately 77% plant death. This suggested that the disease nursery provided a strong selection pressure for resistance to *Rhizoctonia solani* infection and an opportunity to identify a genetic basis for this important trait. Three bulks were sampled for WGS each representing 25 plants (0.7% of the total population). Susceptible individuals (S bulk) were selected with respect to leaf symptoms and confirmed as showing root symptoms as well. Resistant individuals (R bulk) showed no visual leaf or root symptoms (Figure 1). An unselected bulk was also sampled which helped to identify allele frequency changes resulting from selection vs. historical population dynamics and/or genetic drift.

An average of 259,888,506 reads were generated for each of the three bulks, representing an average coverage of 80.3X per sample. The raw reads were trimmed and mapped to the EL10.2 reference genome: 98.1% of bases were retained after filtering and 95% of reads successfully aligned. The aligned reads were used to identify sequence variation across the three populations. A total of 3,235,162 variants were detected, consisting of biallelic, multi-allelic, and structural variants (SV). Biallelic variation accounted for 2,812,301 loci (86.93%) of the total variation and multi-allelic variation accounted for 249,045 (7.70%). Biallelic and multi allelic variation was further categorized by type, including insertions, deletions, single nucleotide polymorphisms (SNP), multi nucleotide polymorphisms (mnp) and complex substitutions (Table 1). The biallelic variation was used for statistical analysis due to its ease in estimating allele frequency and population genomic parameters. Expected heterozygosity (*He*) showed the degree of fixation resulting from selection for a resistance phenotype. A reduction in *He* was observed between the unselected (0.304) and the resistant bulk (0.298). *He* for the susceptible bulk (0.305) was closer to the unselected bulk (Table 1). This is consistent with selection pressure applied and the frequency that resistance was observed in the base EL57 population. We also found 173,817 SVs (5.37%), which were subcategorized as insertions (26,837), deletions (68,922), and putative translocations (78,057) relative to the EL10.2 genome (Table 1).

**TABLE 1:** Summary of variant detection.

	Number	Percent (%)
**Total variants**	3,235,162	100.00%
Biallelic	2,812,301	86.93%
Multiallelic	249,045	7.70%
Structural variant (SV)	173,816	5.37%
*SV insertion*	*26,837*	
*SV deletion*	*68,922*	
*SV translocations*	*78,057*	
**Biallelic**	2,812,301	100.00%
Single nucleotide polymorphism (SNP)	1,939,590	68.97%
Insertion	454,829	16.17%
Deletion	186,790	6.64%
Complex substitution	168,053	5.98%
Multi nucleotide polymorphism (MNP)	63,039	2.24%

**Population**	**Expected heterozygocity (2pq)**	

EL57 unselected bulk	0.304	
EL57 resistant bulk	0.298	
EL57 susceptible bulk	0.305	

**FIGURE 2:**
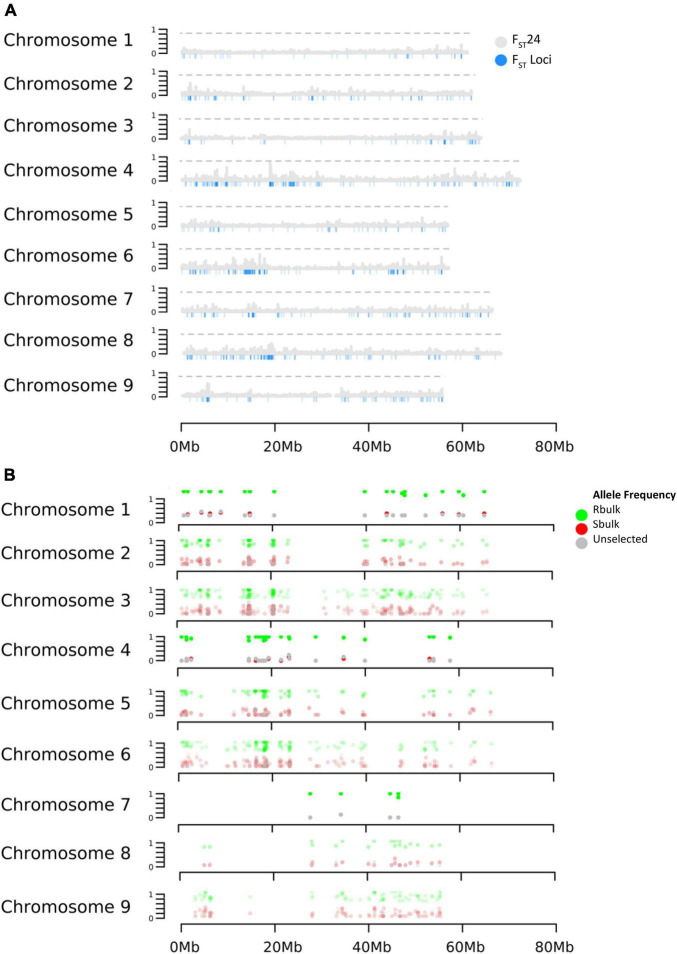
Effects of selection on allele frequency across *B. vulgaris* chromosomes. **(A)** Distribution of F_ST_ 24 and F_ST_ **(B)** Distribution of DeltaAF.

Selection was investigated across the genome using the parameters F_ST_ and DeltaAF. F_ST_ estimated the apportionment of variation in allele frequency between bulks. The empirical distribution of F_ST_ allowed us to assign significance values (*p*-values) to all biallelic loci. At significance levels of *p*< 0.05, *p*< 0.01, and *p*< 0.001. F_ST_ values were equal to 0.22, 0.84, and 0.91 respectively. In total, 1,311 loci were detected with significant F_ST_ (*p* < 0.01). Since F_ST_ shows divergence at a single site, it can be hard to interpret if the divergence is the result of genetic drift or selection. To address this issue, F_ST_ was also calculated in a sliding window (F_ST_24) which considered 24 adjacent variant sites as a single entity. This could reduce noise from genetic drift under the assumption that if selection was acting on a site, linkage disequilibrium would cause adjacent sites to diverge along with the causal variant. As expected, the F_ST_24 analysis reduced the number of significant regions associated with divergence between the resistant and susceptible bulks (Figure 2 and Table 2). Divergence between resistant and susceptible bulks, measured by F_ST_, occurred on all chromosomes but some chromosomes contained more divergent loci than others. It was also noted that divergent sites appeared to be within gene rich regions, and not associated with centromeric or telomeric sequences.

**TABLE 2:** Accumulation of significant loci among chromosomes.

	FST (*p* < 0.01)	FST24 (*p* < 0.01)	DeltaAF
Chromosome 1	314	153	37
Chromosome 2	204	18	32
Chromosome 3	145	0	25
Chromosome 4	79	4	13
Chromosome 5	117	26	14
Chromosome 6	50	0	7
Chromosome 7	235	112	48
Chromosome 8	69	0	5
Chromosome 9	103	52	5

Delta AF was used to test changes in allele frequency between populations selected for resistance and susceptibility to rhizoctonia vs. an unselected population. Our expectation for DeltaAF was that large differences in allele frequency detected between susceptible and resistant bulk would not be found between susceptible and unselected bulks, given the frequency of resistant individuals in the unselected bulk was estimated at 23%. In terms of significant sites, DeltaAF was a more stringent statistic. In total, 186 sites were detected as significant, representing 42 genes. The complete table of all DeltaAF loci are presented in Supplementary Table 2. Comparisons between significant loci produced by F_ST_, F_ST_ 24, and DeltaAF across the chromosomes are present in Table 2.

Significant genomic regions were defined by taking all 1311 F_ST_ values which passed the significance threshold of 0.84 (*p* < 0.01) and looking for physical clusters of significant F_ST_ values within 50 kb of a given locus. This produced a list of regions along the chromosome which were the most diverged between the resistant and susceptible bulks (Table 3). In total 206 regions were identified and the size and magnitude of significance for each region were evaluated. In total 83 of the regions were represented by only one locus with significant F_*ST*_. These appeared less likely to reflect a selective sweep but represent potential functional variants between pools and may contribute to the phenotype. The remaining 43 regions were represented by more than a single locus (3–17 loci) within 50 kb of another significant variant site. The average size of a significant region was 27,624 bp, with a range from 3 to 130,093 bp in length. The complete table with F_ST_ values is presented in Supplementary Table 1. Significant DeltaAF loci were associated with 136 regions across the chromosome and were determined the same way as F_ST_.

**TABLE 3:** Comparison of regions derived from F_ST_ and Delta AF.

	Number of regions	Genes associated with regions	Promoters associated with regions	Average number of loci in regions	Average size of region (bp)
**FST**	206	275	36	6.39	14,998
**DeltaAF**	136	32	10	1.37	3,559

Genes that were associated with significant F_ST_ and DeltaAF values were extracted by using a significance threshold and determining their physical position relative to gene boundaries (5′ URT and 3′ UTR) or the upstream promoter sequences, defined as 3 kb flanking the gene. In total, 1,316 loci with high F_ST_ were associated with 206 regions, containing 311 genes. These could be further subdivided into variants associated with the gene sequences (275) (Supplementary Table 3) and those associated promoter sequences of genes (36) (Supplementary Table 4). In total, DeltaAF recovered 42 genes, and 32 were identified as having high DeltaAF signals within the gene boundaries (Supplementary Table 5) and 10 were identified in putative promoter sequence (3 kb flanking the genes) (Supplementary Table 6). All of the genes associated with Delta AF genes were found within the larger F_ST_ gene set. The total biallelic variant set was analyzed using SNPeff and produced 56,451 annotations predicted to have high consequences on gene function. Only 11 of these coincide with loci determined to have significant F_ST_ or DeltaAF.

**FIGURE 3:**
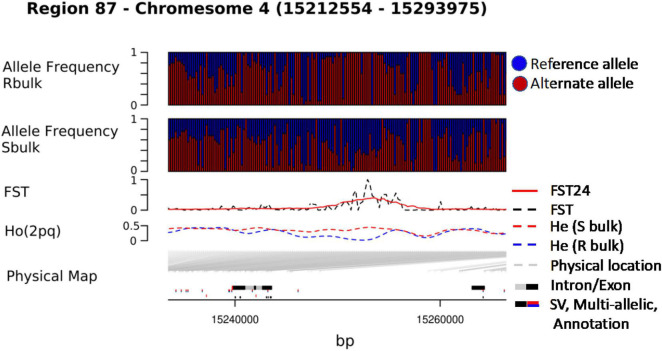
Visualization of chromosome regions on the basis significant F_*ST*_ (*p*-value<0.01).

We combined data from statistical tests for enrichment (e.g., F_ST_ and DeltaAF) with custom visualization tools to inspect regions of significance with respect to the EL10.2 physical map (Figure 3). A preliminary set of 41 candidate regions was generated based on their proximity and effect of genetic variation relative to gene models. We queried publicly available databases and the scientific literature to determine the functional identity of the candidate genes to prioritize targets of future research. This analysis revealed 18 genes with known or putative function in pathogen resistance and six genes likely involved in cell wall metabolism (Table 4). The pathogen defense related genes included multiple representatives from three classes: four chitinases (EL10Ac3g05998, EL10Ac3g06002, EL10Ac3g06003, EL10Ac3g05996), three putative pathogen-responsive Ser/Thr receptor kinases (EL10Ac4g07999, EL10Ac3g06055, EL10Ac3g06056), and two genes involved in defense-associated volatile ester catabolism (EL10Ac6g14646 and EL10Ac3g05812). The cell wall-related genes included five metabolic genes (EL10Ac6g13717, EL10Ac6g13257, EL10Ac5g13023, EL10Ac3g05157, and EL10Ac3g05159) as well as one Myb-related transcription factor EL10Ac1g00142. It is noteworthy that the Peroxidase 5 genes EL10Ac3g05157 and EL10Ac3g05159 are likely a single gene with a transposon inserted into the coding region in the EL10.2 reference genome (the transposon is recorded as EL10Ac3g05158 “Retrovirus-related Pol polyprotein from transposon TNT1–94” in the EL10.2 annotation). Unfortunately, the variant detection strategy employed in this report cannot determine if this peroxidase gene is intact in either the resistant or susceptible bulks. However, the combination of F_ST_, DeltaAF, and visualization of variant positions was able to generate a plausible candidate gene list for further investigation. Potential markers and their significance were reported which could be used for the prediction of resistance (Table 5). Subsequent rounds of the “Select and Sequence” strategy would help to validate the markers generated and inform how genomic prediction might be applied to beet populations segregating for phenotypes of interest.

**TABLE 4:** Candidate genes derived from F_ST_, DeltaAF and proximity to chromosomal regions of high significance.

Data	Chr	Gene	Scaffold	Start	Stop	Strand	Identity
FSTG	1	EL10Ac1g00142	lcl| Scaffold_4	62288988	62293643	−	Myb-related protein Zm1
Region	3	EL10Ac3g05118	lcl| Scaffold_7	2505514	2508049	+	Mitochondrial metalloendopeptidase OMA1
DAF/FSTG	3	EL10Ac3g05157	lcl| Scaffold_7	2964465	2988432	+	Peroxidase 5 {ECO:0000250| UniProtKB:P22195}
Region	3	EL10Ac3g05158	lcl| Scaffold_7	2975394	2980297	−	Retrovirus-related Pol polyprotein from transposon TNT1-94
Region	3	EL10Ac3g05159	lcl| Scaffold_7	2986817	2992340	−	Peroxidase 5 {ECO:0000250| UniProtKB:P22195}
FSTG	3	EL10Ac3g05812	lcl| Scaffold_7	11447524	11463930	−	Probable carboxylesterase 1
FSTG	3	EL10Ac3g05814	lcl| Scaffold_7	11478272	11491769	+	Putative disease resistance protein RGA1
FSTG	3	EL10Ac3g05956	lcl| Scaffold_7	13796358	13808136	−	Cytosolic sulfotransferase 15
Region	3	EL10Ac3g05996	lcl| Scaffold_7	14499893	14502240	+	Endochitinase CH25
Region	3	EL10Ac3g05998	lcl| Scaffold_7	14524198	14525516	+	Endochitinase
Region	3	EL10Ac3g06002	lcl| Scaffold_7	14553565	14555809	+	Endochitinase A
FSTG	3	EL10Ac3g06003	lcl| Scaffold_7	14553822	14558536	−	Chitinase 9
FSTG	3	EL10Ac3g06012	lcl| Scaffold_7	14703412	14714093	−	3beta-hydroxysteroid-dehydrogenase/decarboxylase isoform 3
FSTG	3	EL10Ac3g06014	lcl| Scaffold_7	14744397	14756713	−	Calreticulin-3
Region	3	EL10Ac3g06055	lcl| Scaffold_7	15643877	15645017	+	Wall-associated receptor kinase 1
FSTP	3	EL10Ac3g06056	lcl| Scaffold_7	15666223	15687673	+	Wall-associated receptor kinase 2
Region	4	EL10Ac4g07999	lcl| Scaffold_3	6786108	6793448	−	Probable leucine-rich repeat receptor-like serine/threonine-protein kinase At5g15730
Region	5	EL10Ac5g12121	lcl| Scaffold_2	16531411	16538896	+	zinc-binding in reverse transcriptase
FSTG	5	EL10Ac5g13023	lcl| Scaffold_2	787508	799504	−	Oxysterol-binding protein-related protein 2A
FSTG	6	EL10Ac6g13257	lcl| Scaffold_1	69644352	69648601	−	Endo-1,4-beta-xylanase F1
FSTP	6	EL10Ac6g13717	lcl| Scaffold_1	62821741	62829006	+	Anthocyanidin 3-O-glucoside 2”-O-glucosyltransferase
NA	6	EL10Ac6g14646	lcl| Scaffold_1	22176769	22179875	−	Valencene synthase
FSTG	6	EL10Ac6g15325	lcl| Scaffold_1	7486762	7502133	+	transmembrane protein 184A
FSTG	6	EL10Ac6g15331	lcl| Scaffold_1	7409666	7416329	−	Probable E3 ubiquitin-protein ligase HERC2
Region	6	EL10Ac6g15568	lcl| Scaffold_1	3124396	3127216	+	hypothetical protein
FSTG	8	EL10Ac8g19585	lcl| Scaffold_5	30096389	30106598	+	Cationic amino acid transporter 1
FSTG	3	EL10Ac9g21282	lcl| Scaffold_9	41429473	41431399	−	Vacuolar amino acid transporter 1
Region	5	EL10As14g24073	lcl| Scaffold_2	14271095	14272339	+	Protein SLE2 {ECO:0000303| Ref.3}
FSTP	5	EL10As14g24074	lcl| Scaffold_2	14288341	14294899	+	Dynamin-related protein 1E

*FSTG, significant FST loci within gene; FSTP, significant FST loci within promoter sequence; DAF, significant DeltaAF loci; Region, gene found within significant region; NA, gene found within significant region.*

**TABLE 5:** Marker variation-high consequence (SNPeff), F_ST_, and DeltaAF.

					Allele Frequency					
Data	EL10.2 Scaffold	Position	Ref	Alt	Unseleted	Resistant	Suceptible	FST	EL10.1 Gene ID	Gene Annotation
SNPeff	lcl| Scaffold_1	10,879,102	TGG	TGGG	0.00	0.00	1.00	1.00	EL10Ac6g15089	RNA recognition motif. (a.k.a. RRM, RBD, or RNP domain)
SNPeff	lcl| Scaffold_1	26,110,683	CAA	CAAA	1.00	0.00	0.92	0.85	EL10Ac6g14526	Zinc finger CCHC domain-containing protein 8
SNPeff	lcl| Scaffold_2	56,034,911	GG	GCA	0.88	1.00	0.16	0.72	EL10Ac5g11051	NEP1-interacting protein-like 2;TMhmm_ExpAA:32.25
SNPeff	lcl| Scaffold_4	62,342,250	CTC	CTTG	1.00	0.00	1.00	1.00	EL10Ac1g00136	Carboxymethylenebutenolidase homolog
SNPeff	lcl| Scaffold_7	52,713,203	TA	TGG	0.92	0.00	1.00	1.00	EL10Ac3g07219	Nucleolar protein 10
SNPeff	lcl| Scaffold_8	452,635	TT	TAGGA	0.88	0.00	0.84	0.72	EL10Ac2g02429	Histidine kinase 5
SNPeff	lcl| Scaffold_9	48,328,478	AGA	AGGG	0.92	0.00	1.00	1.00	EL10Ac9g21727	Probable receptor-like serine/threonine-protein kinase At4g34500;TMhmm_ExpAA:23.97
SNPeff	lcl| Scaffold_9	52,891,057	TA	TGGC	0.80	0.00	1.00	1.00	EL10Ac9g22099	GATA transcription factor 12
SNPeff	lcl| Scaffold_9	53,136,509	GAG	GAAC	0.68	1.00	0.16	0.72	EL10Ac9g22117	MADS-box transcription factor 27
DAF/FST	lcl| Scaffold_4	63,029,953	C	T	0.08	1.00	0.00	1.00	EL10Ac6g15080	Protein FAR-RED ELONGATED HYPOCOTYL 3
DAF/FST	lcl| Scaffold_4	63,029,962	A	G	0.08	1.00	0.00	1.00	EL10Ac6g14731	Probable inactive receptor kinase At5g10020;TMhmm_ExpAA:41.51
DAF/FST	lcl| Scaffold_4	53,504,844	T	C	0.00	1.00	0.00	1.00	EL10Ac6g14670	Replication factor C subunit 1
DAF/FST	lcl| Scaffold_4	40,900,804	TGGGGGGGG GGGGGGGA	TGGGGGGGGGG GGGGGGA	0.04	0.96	0.00	0.92	EL10Ac6g14562	Importin-9;TMhmm_ExpAA:27.48
DAF/FST	lcl| Scaffold_4	1,904,674	CA	AG	0.00	0.84	0.00	0.72	EL10Ac6g13913	Sugar transport protein 14
DAF/FST	lcl| Scaffold_4	1,904,696	T	A	0.00	0.88	0.00	0.79	EL10Ac5g12073	Putative disease resistance protein RGA3
DAF/FST	lcl| Scaffold_8	2,814,345	A	G	0.00	1.00	0.00	1.00	EL10Ac5g12061	Transcription factor MYB39
DAF/FST	lcl| Scaffold_8	3,930,082	G	A	0.00	0.88	0.00	0.79	EL10Ac5g11572	Metallothionein
DAF/FST	lcl| Scaffold_7	1,850,367	A	T	0.16	1.00	0.12	0.79	EL10Ac4g08331	Putative ribonuclease H protein At1g65750
DAF/FST	lcl| Scaffold_7	2,965,251	AT	ATACAGT	0.00	1.00	0.00	1.00	EL10Ac4g09188	Guanine deaminase
DAF/FST	lcl| Scaffold_7	2,965,294	C	T	0.00	1.00	0.00	1.00	EL10Ac4g09998	Protein kri1
DAF/FST	lcl| Scaffold_7	14,304,193	GAA	GA	0.00	1.00	0.00	1.00	EL10Ac1g02366	Heptahelical transmembrane protein 4;TMhmm_ExpAA:151.43
DAF/FST	lcl| Scaffold_7	15,181,123	GTAAACTAGTAAC	GGCCAG	0.00	1.00	0.00	1.00	EL10Ac1g02366	Heptahelical transmembrane protein 4;TMhmm_ExpAA:151.43
DAF/FST	lcl| Scaffold_7	15,643,326	A	G	0.08	1.00	0.00	1.00	EL10Ac1g02366	Heptahelical transmembrane protein 4;TMhmm_ExpAA:151.43
DAF/FST	lcl| Scaffold_7	15,665,864	G	A	0.00	1.00	0.00	1.00	EL10Ac1g01440	Small RNA degrading nuclease 5
DAF/FST	lcl| Scaffold_7	15,665,870	GTAAGAAAGTGACAT	GTAAGAAAGTGACATA AGAAAGTGACAT	0.00	1.00	0.00	1.00	EL10Ac1g00071	ABC transporter E family member 2
DAF/FST	lcl| Scaffold_7	16,724,330	GGATC	AGATT	0.00	1.00	0.00	1.00	EL10Ac1g00071	ABC transporter E family member 2
DAF/FST	lcl| Scaffold_7	16,724,391	TATC	AATT	0.00	1.00	0.00	1.00	EL10Ac8g18254	BNR repeat-like domain
DAF/FST	lcl| Scaffold_7	16,724,418	A	C	0.00	1.00	0.00	1.00	EL10Ac8g18254	BNR repeat-like domain
DAF/FST	lcl| Scaffold_7	25,250,059	AGTGGTTGTGGTTGTG GTTGTGGTTGTGGTTG TGGTTGTGGTTGTGGT TGTGGTTGTGGTTGTGG TTGTGGTTG	AGTGGTTGTGGTTGTGG TTGTGGTTGTGGTTGTG GTTGTGGTTGTGGTTGT GGTTGTGGTTG	0.08	0.92	0.04	0.78	EL10Ac8g20189	U4/U6.U5 tri-snRNP-associated protein 2
DAF/FST	lcl| Scaffold_7	52,512,965	TG	GG	0.00	1.00	0.00	1.00	EL10Ac7g17584	Major allergen Pru ar 1
DAF/FST	lcl| Scaffold_3	19,694,86	G	A	0.00	1.00	0.04	0.92	EL10Ac3g05987	Lysine–tRNA ligase;TMhmm_ExpAA:77.60
DAF/FST	lcl| Scaffold_3	15,282,274	GGG	AAA	0.00	1.00	0.00	1.00	EL10Ac3g06032	Hypothetical protein;TMhmm_ExpAA:36.46
DAF/FST	lcl| Scaffold_3	48,281,437	A	C	0.00	1.00	0.00	1.00	EL10Ac3g06055	Wall-associated receptor kinase 1
DAF/FST	lcl| Scaffold_3	56,318,571	TTATATATATATATATATATA TATATATATATATATATATATATA TATATATATATATATAT	TTATATATATATATATA TATATATATATAT	0.04	1.00	0.08	0.85	EL10Ac3g06056	Wall-associated receptor kinase 2;TMhmm_ExpAA:46.88
DAF/FST	lcl| Scaffold_3	60,769,635	G	C	0.00	0.84	0.00	0.72	EL10Ac3g06056	Wall-associated receptor kinase 2;TMhmm_ExpAA:46.88
DAF/FST	lcl| Scaffold_2	35,363,747	GTTTTTTTTTTTTTTG	GTTTTTTTTTTTTTTTTTTTG	0.16	0.96	0.08	0.78	EL10Ac3g06056	Wall-associated receptor kinase 2;TMhmm_ExpAA:46.88
DAF/FST	lcl| Scaffold_2	18,141,331	A	T	0.00	1.00	0.00	1.00	EL10Ac3g06056	Wall-associated receptor kinase 2;TMhmm_ExpAA:46.88
DAF/FST	lcl| Scaffold_2	17,790,729	T	A	0.00	1.00	0.00	1.00	EL10Ac3g06102	Protein FAM136A
DAF/FST	lcl| Scaffold_2	15,093,093	C	T	0.00	1.00	0.00	1.00	EL10Ac3g06102	Protein FAM136A
DAF/FST	lcl| Scaffold_1	54,208,817	AAGGGTTAGGGTTT	AAGGGTTAGGGTTAGGGTTT	0.08	1.00	0.08	0.85	EL10Ac3g06102	Protein FAM136A
DAF/FST	lcl| Scaffold_1	24,987,470	CGGGGGGGGGGGGT	CGGGGGGGGGGGGGGT	0.16	1.00	0.12	0.79	EL10Ac3g06102	Protein FAM136A
DAF/FST	lcl| Scaffold_1	21,263,551	TAAAAAG	TAAAAAAG	0.00	0.88	0.00	0.79	EL10Ac3g06102	Protein FAM136A
DAF/FST	lcl| Scaffold_1	19,398,911	C	A	0.00	1.00	0.00	1.00	EL10Ac3g06102	Protein FAM136A
DAF/FST	lcl| Scaffold_1	19,217,784	G	A	0.00	1.00	0.00	1.00	EL10Ac3g06102	Protein FAM136A
DAF/FST	lcl| Scaffold_1	11,698,071	C	A	0.12	1.00	0.12	0.79	EL10Ac3g06102	Protein FAM136A
DAF/FST	lcl| Scaffold_1	11,468,948	A	T	0.00	1.00	0.00	1.00	EL10Ac3g06102	Protein FAM136A
DAF/FST	lcl| Scaffold_1	2,848,411	GAA	GAAA	0.00	1.00	0.00	1.00	EL10Ac3g05053	Hypothetical protein
DAF/FST	lcl| Scaffold_6	33,124,391	AATATATATATATATAT ATATATATATATA	AATATATATATATATATA TATATATATATATA	0.00	0.92	0.08	0.71	EL10Ac3g06365	Light-mediated development protein DET1
DAF/FST	lcl| Scaffold_5	61,709,065	GT	AC	0.00	1.00	0.00	1.00	EL10Ac3g05157	Peroxidase 5 {ECO:0000250| UniProtKB:P22195}
DAF/FST	lcl| Scaffold_5	61,709,075	G	A	0.08	1.00	0.00	1.00	EL10Ac3g05157	Peroxidase 5 {ECO:0000250| UniProtKB:P22195}
DAF/FST	lcl| Scaffold_5	48,904,484	C	T	0.00	1.00	0.08	0.85	EL10Ac3g07201	Two-component response regulator ARR9
DAF/FST	lcl| Scaffold_5	42,132,982	C	A	0.04	1.00	0.00	1.00	EL10Ac2g02580	Cytochrome b-c1 complex subunit 7
DAF/FST	lcl| Scaffold_5	7,244,134	GCC	GC	0.08	0.88	0.00	0.79	EL10Ac2g02645	Hypothetical protein
DAF/FST	lcl| Scaffold_9	34,647,812	GCTGGACTGGAC	GCTGGACTGGACTGGAC	0.12	1.00	0.12	0.79	EL10Ac9g21035	Exosome component 10
DAF/FST	lcl| Scaffold_9	45,146,825	G	T	0.00	1.00	0.00	1.00	EL10Ac9g21479	Calmodulin-binding transcription activator 1

## Discussion

Identifying the genetic basis of quantitative traits is a longstanding challenge in crop improvement. In this report, we used a select and sequence (SnS) approach to identify contrasting genetic variants between resistant and susceptible bulks drawn from a synthetic breeding population segregating for resistance to seedling rhizoctonia infection. Using pooled sequencing, we estimated population genetic parameters to investigate fixation (He), genome divergence (F_*ST*_) and changes in allele frequency (DeltaAF) resulting from selection and identified candidate genomic variation underlying Rhizoctonia resistance. We generated a list of putative candidate genes by visualizing the population genetic data with respect to the EL10.2 physical map. The candidate gene list was enriched for genes associated with pathogen defense and cell—wall biosynthesis, both of which are plausible components of rhizoctonia resistance. Additional rounds of selection within the EL57 base population or the advancement of generations in the presence of divergent selection could further resolve causal genetic variation and validate the genetic basis of Rhizoctonia resistance in beet.

SnS experiments using segregating populations provide a system to study the underlying genetics of polygenic traits as part of ongoing selection activities within a breeding program. Here we show how pooled sequencing could be used for discovery of key genetic variation when applied to polygenic traits within a population with an extremely broad genetic base. In this case, Rhizoctonia resistance stored within the synthetic EL57 population was derived from EL51, which can be traced to FC701 as the likely source (Panella et al., 2015). Significant signals of divergence as the result of selection were distributed across the genome indicative of a polygenic trait. This is consistent with previous reports of trait heritability (Hecker and Ruppel, 1975). Expected heterozygosity (*He*) estimated using all biallelic sites showed a greater level of fixation in the resistant bulk, suggesting that the genetic background that conditions resistance to Rhizoctonia could be selected and identified within a highly heterozygous population. The fact that only a few resistance sources have been identified even with considerable effort suggests genome informed approaches may be key to characterizing the source under study here as well as identifying new sources for Rhizoctonia resistance in other diverse populations.

F_ST_ and DeltaAF are complementary statistics that were both able to identify the effects of selection across the genome. DeltaAF was a more stringent statistic in terms of the number of significant loci and putative genes detected, as evidenced by all significant DeltaAF loci appearing as a subset of significant F_ST_ loci. It has been shown that generating a null distribution of allele frequency in an unselected population is important for separating signal from noise when detecting selection (Galtier and Duret, 2007). Therefore, DeltaAF could have an advantage in identifying causal variation due to its ability to leverage unselected allele frequencies to further distinguish significant loci resulting from selection, as opposed to differences resulting from historical population dynamics or genetic drift.

The final candidate gene list was strongly enriched for disease resistance genes and cell wall biosynthesis genes, suggesting complementary mechanisms involved in host resistance. To better define the genomic background associated with the putative EL51/FC701 resistance source, we specifically focused on genes which can explain resistance and are documented in the literature with known disease resistance functions, such as pathogen perception, signal transduction and cellular response to pathogens. The wide array of obvious defense genes such as Ser/Thr kinases, resistance gene analogs, chitinases, and peroxidases is in line with our expectations of how plants could defend themselves against a generalist pathogen such as Rhizoctonia. Other members of the candidate gene list do not have established roles in plant defense. These are genes with plausible but speculative roles, such as transcription factors and putative cell membrane-associated proteins. The combination of known defense genes along with additional genes of unknown function provides direction for developing testable hypotheses regarding the mechanisms of defense. In addition to genes involved in host-pathogen interactions, variation in cell wall biosynthesis could make some plants more resistant to infection. Previous studies have identified seedling resistance in comparisons between susceptible (USH20) and resistant (EL51) varieties (Nagendran et al., 2009). Resistant plants showed a durable resistance including the ability to limit the spread of infection beyond the epidermis, including maintenance of cell wall integrity in the presence of pathogen-derived enzymes that varied with respect to plant age. For this reason, identifying numerous cell wall-related genes among our significant loci adds evidence to the importance of cell wall biosynthesis in limiting Rhizoctonia infection, especially at the seedling stage.

In conclusion, this research provides a genomic perspective to seedling Rhizoctonia resistance in beets, a complex polygenic trait with agricultural importance. We think it is a useful exercise to develop methods and generate lists of candidate genes involved with important traits in order to validate results and prioritize candidate variation for functional studies. A better understanding of the limitations of these experiments and our ability to detect significant variation is warranted. The detection of PAV in pooled data is perhaps the most visible limitation of this experiment. If PAV is causal and not represented in the genomic data then we rely on linkage, which is not a strength of pooled sequencing designs. Future experiments should address the ability of pooled assemblies to represent the genomes of populations under investigation with respect to PAV, and whether PAV frequency can be measured. Starting with the best possible catalog of variants for population genetic parameters represents the highest degree of resolution for the identification of causal variation. Select and sequence experiments have the potential to explore the genetic base of beet through the identification of alleles in wild material as well as characterize existing germplasm for agriculturally important traits. Using this approach, beet breeding programs can simultaneously generate markers and improve the genetic base of populations using phenotypic selection.

## Data Availability Statement

The original contributions presented in the study are publicly available. This data can be found here: EL57 Illumina reads for the resistance, susceptible and unselected populations were deposited to NCBI under BioProject (PRJNA563463). The EL10.2 genome assembly (https://genomevolution.org/coge/GenomeInfo.pl?gid=57232). All variant files (.vcf) and files used for visualization are available at (Data Dryad - https://doi.org/10.5061/dryad.3j9kd51kg). All code is available at (https://github.com/BetaGenomeNinja/EL57). This includes bash scripts, Jupyter-note books for python code development, python scripts for data manipulation and pop gen estimators and R code for plotting and visualization of data.

## Author Contributions

JM developed the synthetic EL57 population. PG, JM, and AF conceived and designed the experiments and edited reviewed and approved the final manuscript. JM and PG performed the experiments. PG and AF analyzed the data. PG wrote the draft manuscript. All authors contributed to the article and approved the submitted version.

## Conflict of Interest

The authors declare that the research was conducted in the absence of any commercial or financial relationships that could be construed as a potential conflict of interest.

## Publisher’s Note

All claims expressed in this article are solely those of the authors and do not necessarily represent those of their affiliated organizations, or those of the publisher, the editors and the reviewers. Any product that may be evaluated in this article, or claim that may be made by its manufacturer, is not guaranteed or endorsed by the publisher.
